# Extended Endocrine Therapy and Survival for Breast Cancer Subtypes in Premenopausal Patients

**DOI:** 10.1001/jamanetworkopen.2026.10427

**Published:** 2026-05-04

**Authors:** Carmine Valenza, Yue Zheng, Monica Milano, Pier Paolo Maria Berton Giachetti, Dario Trapani, Elisa Giordano, Lorenzo Guidi, Laura Boldrini, Grazia Castellano, Jalissa Katrini, Bianca Malagutti, Gabriele Antonarelli, Julian D. Etessami, Nadia Bianco, Fabio Conforti, Gregory J. Kirkner, Claudia Sangalli, Kate E. Dibble, Nicola Fusco, Marco Colleoni, Meredith M. Regan, Elisabetta Munzone, Giuseppe Curigliano, Ann H. Partridge

**Affiliations:** 1 Harvard Chan School of Public Health, Harvard University, Boston, Massachusetts; 2 Division of New Drugs and Early Drug Development for Innovative Therapies, European Institute of Oncology, IRCCS, Milan, Italy; 3 Department of Oncology and Hemato-Oncology, University of Milan, Milan, Italy; 4 Department of Medical Oncology, Dana-Farber Cancer Institute, Boston, Massachusetts; 5 Breast Oncology Program, Dana-Farber Brigham Cancer Center, Boston, Massachusetts; 6 Department of Data Science, Dana-Farber Cancer Institute, Boston, Massachusetts; 7 Division of Medical Senology, European Institute of Oncology, IRCCS, Milan, Italy; 8 Division of Medical Oncology, Humanitas Gavazzeni, Bergamo, Italy; 9 Clinical Trial Office, European Institute of Oncology, IRCCS, Milan, Italy; 10 Harvard Medical School, Boston, Massachusetts; 11 Division of Pathology, European Institute of Oncology, IRCCS, Milan, Italy

## Abstract

**Importance:**

Premenopausal patients with node-positive, hormone receptor–positive, early breast cancer derive benefit from extended endocrine therapy (EET) following 5 years of luteinizing hormone–releasing hormone (LHRH) agonist–based treatment. The benefit of EET may differ according to surrogate breast cancer subtypes in postmenopausal patients.

**Objective:**

To evaluate the risk of invasive and distant recurrence across all surrogate breast cancer subtypes among patients with node-positive, hormone receptor–positive early breast cancer who remained premenopausal after completing 5 years of adjuvant therapy with an LHRH agonist who received and did not receive EET.

**Design, Setting, and Participants:**

This multicenter cohort study conducted in the United States and Italy used data from 2 prospectively maintained datasets: the Young Women’s Breast Cancer Study and the European Institute of Oncology Breast Cancer cohort. Eligible patients were diagnosed with early breast cancer at 40 years of age or younger between January 2005 and December 2016, had node-positive hormone receptor–positive disease, and remained premenopausal after 5 years of adjuvant LHRH agonist therapy with no evidence of recurrence. Median (IQR) follow-up was 7.3 (4.9-10.3) years. Data were analyzed June 2025.

**Exposure:**

EET (with tamoxifen monotherapy, LHRH agonist plus tamoxifen, or LHRH agonist plus aromatase inhibitor), irrespective of the duration of EET, measured at study baseline (defined as the first day of the sixth year after the initiation of adjuvant ET).

**Main Outcomes and Measures:**

Invasive breast cancer–free survival and distant recurrence–free survival (DRFS) distributions were estimated using the adjusted Kaplan-Meier method among patients with or without the exposure, weighted through propensity score (PS) weighting analysis, with the scientific approach.

**Results:**

In total, 487 patients were included (median [IQR] age at diagnosis, 37 [35-39] years in the EET group and 37 [33-39] years in the no EET group), and 276 received EET for a median (IQR) duration of 3.7 (2.2-5.0) years. Overall, 89 patients (18%) had luminal A–like disease, 298 (61%) had luminal B–like disease, and 100 (21%) had *ERBB2* (formerly *HER2*)–positive disease. The PS-weighted hazard ratio (HR) for invasive breast cancer–free survival comparing the EET with the no EET group was 0.68 (95% CI, 0.32-1.45) in luminal A–like, 0.63 (95% CI, 0.40-1.00) in luminal B–like/*ERBB2*-negative, and 0.62 (95% CI, 0.21-1.87) in *ERBB2*-positive subgroups. The cause-specific PS-weighted HR for DRFS was 0.25 (95% CI, 0.08-0.75) in luminal A–like, 0.54 (95% CI, 0.32-0.94) in luminal B–like/*ERBB2*-negative, and 0.54 (95% CI, 0.12-2.53) in *ERBB2*-positive subgroups.

**Conclusions and Relevance:**

In this cohort study, a lower estimated risk with EET use was observed across all surrogate breast cancer subtypes. However, the lower estimated risk was greatest among patients with luminal A–like disease, a finding that warrants confirmation in larger, prospective cohorts.

## Key Points

**Question:**

Are there differences in the risks of invasive and distant recurrence across all surrogate breast cancer subtypes among patients with node-positive, hormone receptor–positive breast cancer who remain premenopausal after completing 5 years of adjuvant therapy with a luteinizing hormone–releasing hormone agonist and received or did not receive extended endocrine therapy (EET)?

**Findings:**

In this cohort study that included 487 patients, EET had a lower risk estimate than no EET for invasive breast cancer–free survival among patients with luminal A–like, luminal B–like, or *ERBB2*-positive disease.

**Meaning:**

A lower estimated risk with EET was observed across all surrogate breast cancer subtypes.

## Teaser

This cohort study assesses the risk of invasive and distant recurrence among patients with node-positive, hormone receptor–positive early breast cancer who did and did not receive extended endocrine therapy.

## Introduction

Among young women (aged ≤40 years at diagnosis), breast cancer represents the most common cause of cancer-related morbidity and mortality.^1^ Younger age at diagnosis is an independent adverse prognostic factor, particularly in patients with hormone receptor–positive early breast cancer.^2,3^ In this population compared with an older population, tumors are often detected at a more advanced stage, are not routinely detected through screening, and are more frequently characterized by an intrinsic aggressive biology.^4^

Members of our team previously conducted a cohort study of 501 patients with node-positive, hormone receptor–positive early breast cancer who remained premenopausal after 5 years of receiving adjuvant endocrine therapy (ET) with a luteinizing hormone–releasing hormone (LHRH) agonist.^5^ That study showed that the extension of ET (either with tamoxifen alone or by continuing an LHRH agonist plus an oral ET agent) was associated with lower rates of invasive and distant recurrence at a median (IQR) follow-up of 7.3 (4.8-10.3) years after the initial 5-year treatment period.

Despite decades-long risk of breast cancer recurrence, more than 70% of premenopausal patients with node-positive, hormone receptor–positive early breast cancer have no evidence of recurrence 10 years after completing 5 years of LHRH agonist–based ET. Since this treatment is associated with persistent adverse events that could substantially affect quality of life (ie, vasomotor symptoms, psychosocial and sexual concerns), refining the selection of candidates for extended ET (EET) is critical.^6,7^

Growing evidence in postmenopausal women indicates that patterns of recurrence differ across luminal breast cancer subtypes, whether defined by intrinsic molecular profiling (PAM50) or by immunohistochemical surrogate markers.^8,9,10,11^ Luminal B–like tumors, defined by higher proliferation and lower differentiation, are associated with poorer outcomes and a recurrence hazard that is elevated during the first 5 years after diagnosis and declines thereafter, paralleling the early risk pattern observed in hormone receptor–negative disease. In contrast, luminal A–like tumors, which show greater dependence on estrogen receptor signaling, exhibit a lower but more constant hazard of recurrence over time, which becomes approximately comparable to that of luminal B–like disease beyond the first 5 years. Similarly, in women with high-risk *ERBB2* (formerly *HER2*)–positive breast cancer, the risk of recurrence is highest in the first few years after diagnosis, with a pronounced early peak, especially among patients with hormone receptor–negative disease.^12,13^ By contrast, in the subgroup of women with triple-positive breast cancer, recurrences accumulate more gradually and persist up to 10 years after diagnosis, albeit the risk is still mostly concentrated within the first 5 years, such as among patients with luminal B–like/*ERBB2*-negative disease.

On the basis of these observations, several studies investigating postmenopausal patients with early breast cancer evaluated the benefit of EET according to surrogate or intrinsic breast cancer subtypes, showing a higher benefit in the luminal A–like subgroup.^10,11,14^ However, no corresponding data are available for premenopausal patients, for whom patterns of recurrence and benefits of EET may differ. The aim of the current analysis was to evaluate the risk of invasive and distant recurrence among patients with node-positive, hormone receptor–positive early breast cancer who remained premenopausal after completing 5 years of adjuvant therapy with an LHRH who received or did not receive EET across all surrogate breast cancer subtypes.

## Methods

### Study Design and Participants

We conducted an international, multicenter cohort study analysis using 2 prospectively maintained datasets: the Young Women’s Breast Cancer Study (YWS) and the European Institute of Oncology (IEO) breast cancer cohort.^15^ All participants provided written informed consent to enter the cohorts. The current joint analysis was approved by the IEO institutional review board and was conducted in accordance with the principles of the Declaration of Helsinki^16^ and Good Clinical Practice guidelines. The study is in compliance with the European General Data Protection Regulation, the US Health Insurance Portability and Accountability Act, and applicable federal and state privacy regulations for data protection and privacy. Results were reported following the Strengthening the Reporting of Observational Studies in Epidemiology (STROBE) reporting guideline.

We included women diagnosed with early breast cancer who were 40 years of age or younger between 2005 and 2016 and underwent breast surgery for tumors of ductal, lobular, or ductolobular histologic results. Eligible participants had node-positive, nonmetastatic disease (defined as pT-any, pN1-3, and cM0 using the American Joint Committee on Cancer TNM staging system [eighth edition]), and were classified as hormone receptor–positive/*ERBB2* any subtype (estrogen receptor or progesterone receptor [PgR] ≥1% by immunohistochemistry), according to the American Society of Clinical Oncology (ASCO) and College of American Pathologists (CAP) guidelines.^17,18^ Patients were required to have completed 5 years of LHRH agonist–based adjuvant ET with no evidence of distant or locoregional recurrence at that time. They also needed to remain premenopausal following the completion of the first 5 years of adjuvant ET, based on at least 1 of the following criteria: age younger than 45 years (to ensure EET occurring within 50 years of age); plasma estradiol levels in the premenopausal range within 6 months after discontinuation of treatment with the LHRH agonist (with or without receipt of ET, such as tamoxifen, at the time of this blood test); and recovery of menstruation after discontinuation of treatment with the LHRH agonist.

Patients were excluded if they had undergone bilateral oophorectomy or radiotherapy ovarian ablation or had a history of invasive breast cancer prior to the index diagnosis. Patients with *ERBB2*-negative disease without information about PgR and Ki-67 protein status were also excluded from this analysis.

Surrogate breast cancer subtypes were defined as follows: luminal A–like (Ki-67 <20%, and PgR ≥20%, and histologic grade 1-2, and *ERBB2*-negative), luminal B–like/*ERBB2*-negative (Ki-67 ≥20%, or PgR <20%, or histologic grade 3; and *ERBB2*-negative), and *ERBB2*-positive (*ERBB2*-positive per ASCO and CAP guidelines).^19^

### Exposure and Outcomes

The exposure was the initiation of EET (with tamoxifen monotherapy, LHRH agonist plus tamoxifen, or LHRH agonist plus aromatase inhibitor [AI]), irrespective of the treatment duration of EET, measured at study baseline. Study baseline was defined as the first day of the sixth year after the initiation of adjuvant ET. Study end points included (1) invasive breast cancer–free survival, defined as the time from the study baseline to the occurrence of ipsilateral invasive breast cancer recurrence, contralateral invasive breast cancer recurrence, local-regional invasive breast cancer recurrence, distant breast cancer recurrence, or death, whichever occurred first^20^; and (2) distant recurrence–free survival (DRFS), measured as the time from the study baseline to distant breast cancer recurrence as the first breast cancer event, or death, whichever occurred first.

### Statistical Analysis

Descriptive statistics were used to analyze patients’ characteristics. Continuous variables are expressed as the median and IQR. Categorical variables are expressed as numbers and percentages.

Invasive breast cancer–free survival and DRFS distributions were estimated using the adjusted Kaplan-Meier method among patients with or without the exposure, weighted through the propensity score (PS) weighting analysis (with stabilized weights) for the following a priori defined covariates (scientific approach)^21^: cohort (YWS vs IEO), age at diagnosis, tumor histologic findings (ductal vs lobular or ductolobular), tumor dimension (pT1 vs pT2 vs pT3/4), nodal status (pN1 vs pN2/pN3), surrogate breast cancer subtype (luminal A–like vs luminal B–like/*ERBB2*-negative vs *ERBB2*-positive breast cancer), type of adjuvant ET received during the first 5 years (LHRH agonist plus AI vs LHRH agonist plus tamoxifen or LHRH agonist alone), and receipt of adjuvant chemotherapy (yes vs no).

To account for the potential effect on distant recurrences of an ET switch or reinitiation after ipsilateral invasive breast tumor recurrence, contralateral invasive breast tumor recurrence, and local-regional invasive recurrence, DRFS was analyzed with the cause-specific hazard ratio (HR) approach. Invasive breast cancer–free survival and DRFS distributions were described according to the surrogate breast cancer subtypes.

Analyses were conducted June 2025. We used SAS, version 9.4 (SAS Institute Inc), for all analyses.

## Results

Overall, 487 patients were eligible for this analysis. Among them, 276 (57%) received EET and 211 (43%) underwent follow-up after 5 years of ET, including with an LHRH agonist. Demographic and clinical data are presented in Table 1 (the PS-weighted analysis is reported in the eTable in Supplement 1). The median (IQR) age at diagnosis was 37 (35-39) years in the EET group and 37 (33-39) years in the no EET group. Overall, 89 patients (18%) had luminal A–like disease, 298 (61%) luminal B–like/*ERBB2*-negative disease, and 100 (21%) *ERBB2*-positive disease. Compared with the group without EET, the EET group included a greater percentage of patients with more advanced stage of disease at diagnosis or more aggressive biology, as indicated by higher proportions of pT3/pT4 stage (13% vs 7%), pN2/pN3 stage (37% vs 27%), and luminal B–like/*ERBB2*-negative disease (63% vs 58%).

**Table 1.** Characteristics of Patients in the Study Population, by Endocrine Therapy Duration

Characteristic	Extended endocrine therapy (n = 276)	No extended endocrine therapy (n = 211)
Age at diagnosis, median (IQR), y	37 (35-39)	37 (33-39)
Dataset, No. (%)		
IEO	273 (99)	210 (99)
YWS	3 (1)	1 (<1)
Histologic examination findings, No. (%)		
Ductal	253 (92)	201 (95)
Lobular	12 (4)	6 (3)
Mixed	11 (4)	4 (2)
Pathologic tumor classification, No. (%)		
pT1	101 (37)	87 (41)
pT2	137 (50)	110 (52)
pT3-4	38 (13)	14 (7)
Pathologic node classification, No. (%)		
pN1	175 (63)	155 (73)
pN2	61 (22)	35 (17)
pN3-4	40 (15)	21 (10)
Grade, No. (%)		
1	7 (3)	8 (4)
2	131 (47)	112 (53)
3	137 (50)	90 (43)
NA	1 (<1)	1 (<1)
Estrogen receptor, median (IQR), %	90 (80-95)	90 (80-95)
Progesterone receptor, median (IQR), %	70 (20-90)	75 (20-90)
Ki-67, median (IQR), %	25 (21-35)	24 (18-34)
Surrogate subtype, No. (%)		
Luminal A–like	47 (17)	42 (20)
Luminal B–like/*ERBB2*-negative	175 (63)	123 (58)
*ERBB2*-positive	54 (20)	46 (22)
Endocrine therapy during the first 5 y, No. (%)		
LHRH agonist plus tamoxifen	179 (65)	163 (77)
LHRH agonist plus AI	95 (34)	45 (21)
LHRH agonist only	2 (1)	3 (2)
Previous chemotherapy	212 (77)	148 (70)
Anthracyclines and taxanes or CMF	105 (38)	54 (26)
Anthracyclines	84 (30)	83 (39)
CMF or taxanes or other	23 (8)	11 (5)

Abbreviations: AI, aromatase inhibitor; CMF, cyclophosphamide, methotrexate, and fluorouracil; IEO, European Institute of Oncology; LHRH, luteinizing hormone–releasing hormone; NA, not available; YWS, Young Women’s Breast Cancer Study.

During the first 5 years of adjuvant ET, 179 patients (65%) in the EET group and 163 patients (77%) in the group without EET received treatment with an LHRH agonist plus tamoxifen, and 95 patients (34%) in the EET group and 45 patients (21%) in the group without EET received treatment with an LHRH agonist plus AI. In the EET group, 212 patients (77%) received chemotherapy compared with 148 patients (70%) in the group without EET. In both groups, over 90% of patients with *ERBB2*-positive disease received adjuvant trastuzumab.

In the EET group, the median (IQR) duration of EET was 3.7 (2.2-5.0) years. Of 276 patients, 129 (47%) received EET with tamoxifen monotherapy for a median (IQR) duration of 4.0 (2.0-5.0) years. Of 276 patients, 147 (53%) received EET with LHRH agonist plus tamoxifen or an AI for a median (IQR) duration of 3.5 (2.3-5.0) years.

**Figure 1.**
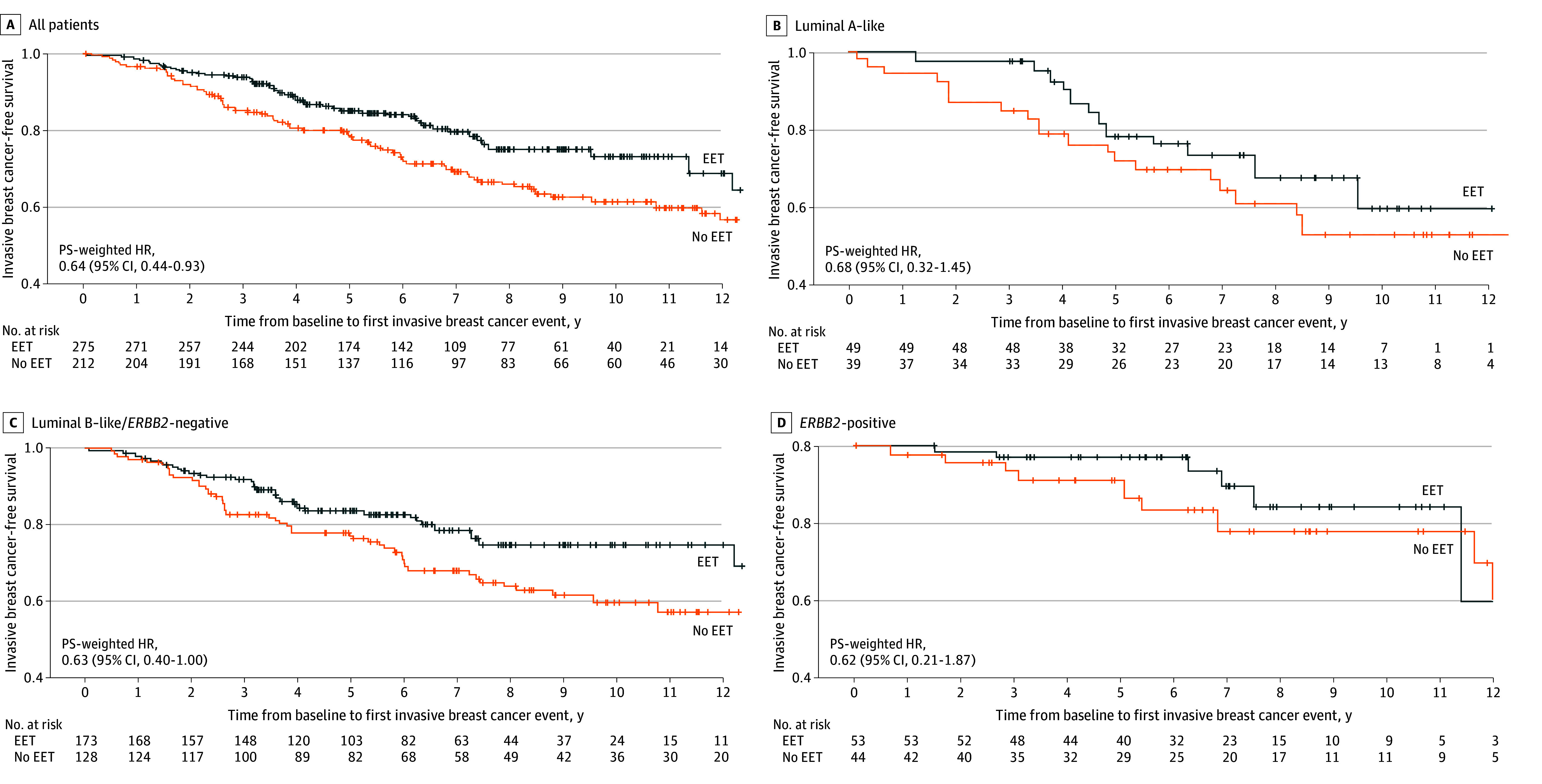
Adjusted Kaplan-Meier Curves of Invasive Breast Cancer–Free Survival by Receipt of Extended Endocrine Therapy (EET) After Propensity Score (PS)-Weighted Analysis Patients had node-positive, hormone receptor–positive early breast cancer and completed 5 years of luteinizing hormone–releasing hormone agonist–based adjuvant endocrine therapy. HR indicates hazard ratio.

After a median (IQR) follow-up of 7.3 (4.9-10.3) years, 52 and 71 invasive breast cancer–free survival events occurred in the EET and no EET groups, respectively (Table 2). The PS-weighted HRs for invasive breast cancer–free survival comparing the EET and no EET groups were 0.64 (95% CI, 0.44-0.93) among all patients and 0.68 (95% CI, 0.32-1.45) in luminal A–like, 0.63 (95% CI, 0.40-1.00) in luminal B–like/*ERBB2*-negative, and 0.62 (95% CI, 0.21-1.87) in *ERBB2*-positive subgroups (Figure 1). In the EET and no EET groups, respectively, the 5-year PS-weighted invasive breast cancer–free survival rates were 85% (95% CI, 80%-89%) and 79% (95% CI, 72%-84%) among all patients; 78% (95% CI, 62%-88%) and 72% (95% CI, 55%-83%) among patients with luminal A–like disease; 84% (95% CI, 77%-89%) and 77% (95% CI, 68%-84%) among patients with luminal B–like disease; and 97% (95% CI, 86%-99%) and 91% (95% CI, 77%-97%) among patients with *ERBB2*-positive disease.

**Table 2.** First Events in Invasive Breast Cancer–Free Survival and Safety Events, by Endocrine Therapy Duration

Event	Events, No. (%)	
	Extended endocrine therapy (n = 276)	No extended endocrine therapy (n = 211)
**All patients**		
No.	276	211
Invasive breast cancer–free survival (first event)	52 (19)	71 (34)
Ipsilateral invasive breast tumor recurrence	5 (2)	11 (5)
Contralateral invasive breast tumor recurrence	14 (5)	8 (4)
Local-regional invasive recurrence	6 (2)	8 (4)
Distant recurrence	27 (10)	43 (21)
Death	0	1 (<1)
**Luminal A**–**like**		
No.	47	42
Invasive breast cancer–free survival (first event)	12 (26)	18 (43)
Ipsilateral invasive breast tumor recurrence	2 (4)	1 (2)
Contralateral invasive breast tumor recurrence	3 (6)	2 (5)
Local-regional invasive recurrence	3 (6)	2 (5)
Distant recurrence	4 (9)	12 (29)
Death	0	1 (2)
**Luminal B**–**like/*ERBB2*-negative**		
No.	175	123
Invasive breast cancer–free survival (first event)	36 (21)	44 (36)
Ipsilateral invasive breast tumor recurrence	3 (2)	8 (7)
Contralateral invasive breast tumor recurrence	8 (5)	5 (4)
Local-regional invasive recurrence	3 (2)	4 (3)
Distant recurrence	20 (11)	27 (22)
Death	0	0
***ERBB2*-positive**		
No.	54	46
Invasive breast cancer–free survival (first event)	6 (11)	9 (20)
Ipsilateral invasive breast tumor recurrence	0	2 (4)
Contralateral invasive breast tumor recurrence	3 (6)	1 (2)
Local-regional invasive recurrence	0	2 (4)
Distant recurrence	3 (6)	4 (9)
Death	0	0

**Figure 2.**
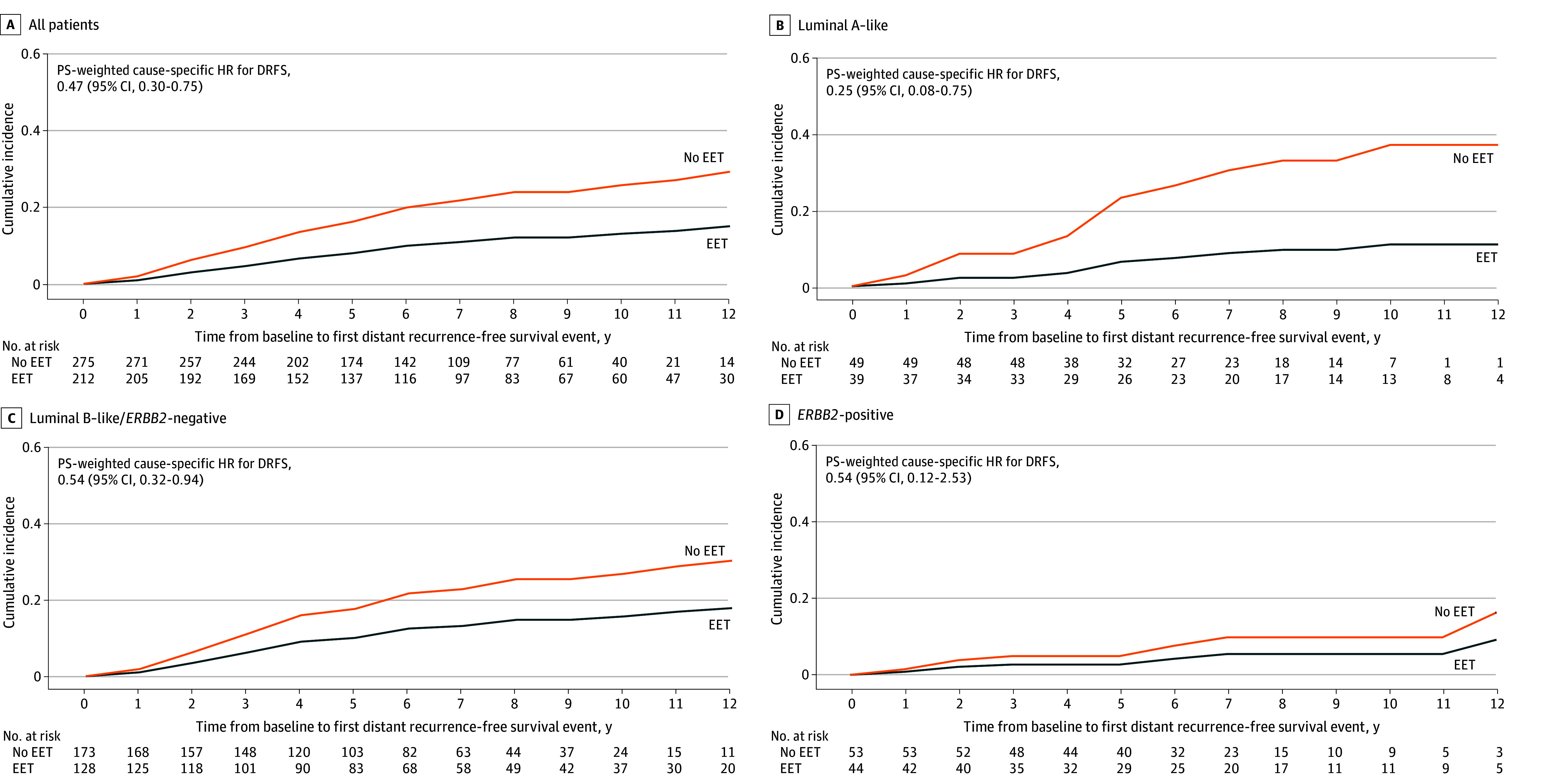
Adjusted Cumulative Incidence Curves of Distant Recurrence–Free Survival (DRFS) by Receipt of Extended Endocrine Therapy (EET) After Propensity Score (PS)-Weighted Analysis Patients had node-positive, hormone receptor–positive early breast cancer and completed 5 years of luteinizing hormone–releasing hormone agonist–based adjuvant endocrine therapy. HR indicates hazard ratio.

A total of 27 and 43 DRFS events occurred in the EET and no EET groups, respectively, as the first event. The PS-weighted cause-specific HR for DRFS comparing the EET and no EET groups was 0.47 (95% CI, 0.30-0.75) in all patients and 0.25 (95% CI, 0.08-0.75) in luminal A–like, 0.54 (95% CI, 0.32-0.94) in luminal B–like/*ERBB2*-negative, and 0.54 (95% CI, 0.12-2.53) in *ERBB2*-positive subgroups (Figure 2). In the EET and no EET groups, respectively, the 5-year PS-weighted cumulative incidence rates of distant recurrence were 8% (95% CI, 6%-11%) and 16% (95% CI, 12%-23%) among all patients; 6% (95% CI, 2%-18%) and 23% (95% CI, 13%-42%) among patients with luminal A–like disease; 10% (95% CI, 7%-15%) and 18% (95% CI, 12%-26%) among patients with luminal B–like disease; and 3% (95% CI, 1%-9%) and 5% (95% CI, 2%-16%) among patients with *ERBB2*-positive disease.

## Discussion

In this secondary analysis, lower estimated risk of invasive and distant recurrence was observed in premenopausal patients with node-positive, hormone receptor–positive early breast cancer who received EET across all surrogate subtypes. The lower estimated risk was greatest among patients with luminal A–like disease (defined by a Ki-67 index below 20%, PgR expression of 20% or higher, histologic grade 1 or 2, and *ERBB2* negativity). Although these patients had a lower overall risk of recurrence than those with luminal B–like tumors, their hazard of recurrence remained more constant over time.^8,9,10,11^ As a result, they may derive proportionally greater benefit from extending ET beyond 5 years, a period during which the recurrence hazard for luminal B–like disease progressively declines, as shown by several subanalyses of clinical trials conducted in the postmenopausal setting.^8,9,10,11^

Luminal A–like disease may be intrinsically more sensitive to ET manipulations than the other subtypes. In the MA.17^14^ and GIM-4^22^ trials, the benefit of extended aromatase inhibition appeared to be confined largely to patients with PgR-positive tumors, a feature characteristic of the luminal A–like subtype. Consistently, the Early Breast Cancer Trialists’ Collaborative Group patient-level meta-analysis including 22 031 postmenopausal women already treated with at least 5 years of ET showed a higher benefit associated with EET for AI use among patients with well-differentiated tumors and PgR-positive disease.^23^ An exploratory analysis of the DATA trial^10^ (treatment with 6 vs 3 years of anastrozole after 2 to 3 years of tamoxifen receipt for postmenopausal patients with early breast cancer) provided similar findings. Patients with luminal A–like tumors derived substantial benefit from treatment extension (subdistribution HR for DRFS, 0.51 [95% CI, 0.30-0.88]), whereas no advantage was observed among patients with luminal B–like disease (subdistribution HR, 2.09 [95% CI, 0.96-4.53]).

The discrepancy observed for luminal B–like disease between our analysis and the DATA trial may reflect differences in patient selection, subtype definitions, and their resulting distribution. In the DATA subanalysis, the different luminal B–like definition (ER positivity combined with either PgR negativity, *ERBB2* positivity, or a Ki-67 ≥14%) together with the inherently less aggressive biology of postmenopausal tumors led to a substantially lower proportion of patients classified as having luminal B–like disease (37% overall and 34% in the node-positive subgroup), compared with the 61% reported in our cohort. Furthermore, patients included in our cohort were highly selected according to relatively young age at diagnosis, positive nodal status, and treatment received during the first 5 years. This finding aligns with a biomarker analysis from the SOFT trial,^24^ which reported that the proportion of patients with luminal A disease defined by PAM50 was 53% among premenopausal women younger than 40 years and 75% among those older than 40 years.

Exploratory biomarker analyses using genomic assays that recapitulate intrinsic biology and luminal features (PAM50 and the 70-gene MammaPrint signature) similarly showed that postmenopausal patients with less aggressive early breast cancer and a lower risk of recurrence (such as those with luminal A or a MammaPrint low-risk tumor) derived greater benefit from EET than patients with more aggressive and highly proliferative disease.^11,25,26^ Studies evaluating the predictive value of genomic assays (such as PAM50, 70-gene MammaPrint, EndoPredict, and Breast Cancer Index) to guide EET in premenopausal patients are lacking. Such evidence may improve the identification of patients most likely to derive benefit and the treatment optimization of patients experiencing persistent adverse events affecting quality of life. Pending these data, surrogate intrinsic subtypes based on immunohistochemistry remain subject to variability in biomarker assessment and uncertainty regarding optimal cutoffs, particularly for Ki-67 and PgR expression. Such methodological heterogeneity may influence subtype classification and treatment effect estimates.

Our findings also suggested a lower estimated risk of invasive and distant recurrence with EET among women with high-risk, *ERBB2*-positive breast cancer, a setting in which this strategy remains debated.^27,28,29^ In clinical practice, decisions regarding ET in this subgroup should aim to optimize outcomes, including consideration of ET extension based on individual clinical risk, although the precise magnitude of the lower estimated risk remains uncertain and could not be fully assessed in our study due to the small size. These findings underscore the need for more targeted investigations and for continued refinement of treatment strategies with modern therapies in this population. A future direction is the validation of blood-based biomarkers, including circulating tumor cells and circulating tumor DNA, which have been associated with late distant recurrences in patients with high-risk hormone receptor–positive early breast cancer and may ultimately inform decisions about treatment extension.^30,31^

### Strengths and Limitations

This study has several strengths. One strength is that it examines the potential role of biomarkers to guide the selection of premenopausal patients who are node-positive for EET, a setting in which evidence is scarce and in which randomization to EET vs no EET would no longer be ethically acceptable. Another strength is that the analysis included patients from 2 prospective cohorts. However, their relative contribution was unbalanced because of differences in the use of an LHRH agonist during the first 5 years of therapy (likely reflecting regional practice patterns at the time the cohorts were assembled) and in the availability of PgR and Ki-67 data, although most assessments were performed at a single specialized breast pathology center (IEO), ensuring consistency in biomarker evaluation.

This study also has limitations. Data on race and ethnicity were not collected. Additionally, this was a secondary, hypothesis-generating study without sufficient power for definitive comparisons, although the overall findings are consistent with results reported in the postmenopausal setting. Indeed, the limited sample size and the small number of patients within each subtype restricted our ability to evaluate interactions between outcomes and surrogate subtypes, and the 95% CIs of the PS-weighted HRs mostly included the HR point estimates of the other subgroups. Larger, adequately powered studies are needed to validate these observations and to determine whether specific subtypes derive differential benefit from treatment extension.

## Conclusions

In this cohort study, a lower estimated risk of invasive and distant recurrence across all breast cancer surrogate subtypes was observed among patients with node-positive, hormone receptor–positive early breast cancer who remained premenopausal after 5 years of LHRH agonist-based treatment who received EET. Notably, although subgroup differences should be interpreted with caution, the lower estimated risk with EET in terms of DRFS may be greater for patients with luminal A–like disease, a finding that warrants confirmation in larger prospective cohorts.

## Notes

Supplement 1. **eTable.** Characteristics of patients with node-positive, hormone receptor-positive early breast cancer who completed 5 years of LHRH agonist-based adjuvant endocrine therapy and were assessed for extended endocrine therapy use, after propensity score weighting

Supplement 2.
Data Sharing Statement
