# Supplementary material for: Extended Endocrine Therapy and Survival for Breast Cancer Subtypes in Premenopausal Patients
Source: JAMA Netw Open. 2026 May 4;9(5):e2610427. doi: 10.1001/jamanetworkopen.2026.10427 (PMC13139950; doi:10.1001/jamanetworkopen.2026.10427)
Supplement: Supplement 1. — eTable. Characteristics of patients with node-positive, hormone receptor-positive early breast cancer who completed 5 years of LHRH agonist-based adjuvant endocrine therapy and were assessed for extended endocrine therapy use, after propensity score weighting [file jamanetwopen-e2610427-s001.pdf]

Supplementary Online Content

Valenza C, Zheng Y, Milano M, et al. Extended endocrine therapy and survival for breast cancer subtypes in premenopausal patients. *JAMA Netw Open*. 2026;9(5):e2610427. doi:10.1001/jamanetworkopen.2026.10427

eTable. Characteristics of patients with node-positive, hormone receptor-positive early breast cancer who completed 5 years of LHRH agonist-based adjuvant endocrine therapy and were assessed for extended endocrine therapy use, after propensity score weighting

This supplementary material has been provided by the authors to give readers additional information about their work.

© 2026 Valenza C et al. *JAMA Network Open*.

eTable. Characteristics of patients with node-positive, hormone receptor-positive early breast cancer who completed 5 years of LHRH agonist-based adjuvant endocrine therapy and were assessed for extended endocrine therapy use, after propensity score weighting*

**Characteristic**	**Extended endocrine therapy (N=275)**	**No extended endocrine therapy (N=212)**
Age at diagnosis, median (IQR)	37 (34-39)	37 (34-39)
Dataset		
IEO, n (%)	273 (99)	210 (99)
YWS, n (%)	2 (1)	2 (1)
Histology		
Ductal, n (%)	257 (93)	197 (93)
Lobular, n (%)	9 (3)	7 (3)
Mized, n (%)	10 (4)	8 (4)
pT		
pT1, n (%)	108 (39)	84 (40)
pT2, n (%)	137 (50)	103 (49)
pT3-4, n (%)	30 (11)	24 (12)
pN		
pN1, n (%)	186 (68)	143 (68)
pN2, n (%)	55 (20)	42 (20)
pN3-4, n (%)	34 (12)	26 (12)
Surrogate subtype		
Luminal A-like, n (%)	49 (18)	39 (19)
Luminal B-like/HER2-negative, n (%)	173 (63)	128 (61)
HER2-positive, n (%)	53 (19)	44 (21)
Endocrine therapy during the first 5 years		
LHRH-agonist plus tamoxifen, n (%)	194 (70)	148 (70)
LHRH-agonist plus AI, n (%)	80 (29)	61 (29)
LHRH-agonist only, n (%)	2 (1)	3 (1)
Previous chemotherapy, n (%)	204 (74%)	156 (74%)

* The propensity score was calculated based on the following variables: dataset, age at diagnosis, histology, tumor stage, nodal stage, surrogate subtype, type of endocrine therapy during the first 5 years, receipt of chemotherapy.

Legend: AI, aromatase inhibitor, IEO, European Institute of Oncology; IQR, interquartile range; n, number; NA, not available; YWS, Young Women's Breast Cancer Study

© 2026 Valenza C et al. *JAMA Network Open.*
