# Supplementary material for: Extended Endocrine Therapy and Survival for Breast Cancer Subtypes in Premenopausal Patients
Source: JAMA Netw Open. 2026 May 4;9(5):e2610427. doi: 10.1001/jamanetworkopen.2026.10427 (PMC13139950; doi:10.1001/jamanetworkopen.2026.10427)
Supplement: Supplement 2. — Data Sharing Statement [file jamanetwopen-e2610427-s002.pdf]

# Data Sharing Statement

Valenza. Extended Endocrine Therapy and Survival for Breast Cancer Subtypes in Premenopausal Patients. *JAMA Netw Open*. Published May 04, 2026.
doi:10.1001/jamanetworkopen.2026.10427

**Data**
**Data available:** No
